# Emergency Preparedness for Local Anesthetic Systemic Toxicity in Dental Practice: Dentists’ Knowledge, Awareness, and Institutional Availability of Lipid Emulsion Therapy

**DOI:** 10.3390/healthcare14142033

**Published:** 2026-07-08

**Authors:** Elif Pınar Bakır, Mehmet Salık, Şeyhmus Bakır

**Affiliations:** 1 Department of Restorative Dentistry, Faculty of Dentistry, Dicle University, Diyarbakır 21280, Türkiye; elifpinarbakir@gmail.com; 2 Department of Restorative Dentistry, Faculty of Dentistry, Batman University, Batman 72060, Türkiye; seyhmusbakir@gmail.com

**Keywords:** local anesthetic systemic toxicity (LAST), lipid emulsion therapy, awareness level, local anesthetics

## Abstract

**Highlights:**

**What are the main findings?**

- Detailed knowledge of lipid emulsion administration steps and dosing protocols was reported by only 0.5% of the participating dentists.
- Most participants either did not know whether lipid emulsion was available at their institution or reported that it was unavailable.

**What are the implications of the main findings?**

- Practice-oriented education on the prevention and management of local anesthetic systemic toxicity should be strengthened in undergraduate and continuing dental education.
- Dental institutions should review lipid emulsion availability, emergency response protocols, and staff preparedness.

**Abstract:**

Objective: This study aimed to evaluate the knowledge and clinical awareness of local anesthetic systemic toxicity (LAST), preventive practices, knowledge of lipid emulsion therapy, and institutional availability among dentists actively practicing in Türkiye, and to examine the demographic and professional factors associated with knowledge level. Methods: This descriptive cross-sectional study was conducted using a 15-item online questionnaire developed by the researchers. The analyses included 369 dentists actively practicing in Türkiye. Data were analyzed using descriptive statistics, the Kruskal–Wallis test, Dunn–Bonferroni pairwise comparisons, Spearman rank correlation, and multinomial logistic regression analysis. Results: Among the participants, 45.8% reported having basic knowledge of LAST, whereas only 2.7% reported detailed knowledge, including the management steps. Although 40.1% stated that they calculated the local anesthetic dose according to the patient’s body weight, only 3.3% reported preparing an emergency response plan for LAST, and 2.2% indicated that they were prepared to use treatment options such as lipid emulsion. Regarding lipid emulsion therapy, 59.1% of participants had low knowledge and 24.4% had superficial knowledge; only 0.5% reported detailed knowledge of the administration steps and dosing protocol. In terms of institutional availability, 45.0% did not know whether lipid emulsion was available at their institution, 40.9% reported that it was unavailable, and 14.1% reported that it was available. Knowledge levels differed according to professional status; however, the effect size was small (H(2) = 13.129; *p* = 0.001; ε^2^ = 0.030). No statistically significant association was found between years of professional experience and knowledge level (ρ = 0.020; *p* = 0.702). Conclusions: Although dentists’ self-reported awareness of LAST varied, detailed knowledge of the administration steps and dosing protocol for lipid emulsion therapy, as well as institutional preparedness, remained limited. The findings suggest that strengthening practice-oriented education on the prevention and management of LAST and reviewing lipid emulsion availability and emergency response protocols in clinical institutions may be beneficial.

## 1. Introduction

Local anesthesia is a fundamental component of effective pain control in a wide range of dental procedures, including tooth extraction, cavity preparation, root canal treatment, abscess drainage, and minor oral surgical interventions [1]. However, local anesthetic systemic toxicity (LAST) may occur when blood concentrations of the drug exceed safe limits as a result of overdose, repeated administration, or inadvertent intravascular injection of the local anesthetic agent [2]. Although LAST is rare, it is a serious and potentially life-threatening complication that can affect both the central nervous and cardiovascular systems [3,4]. In addition to the administered dose, injection technique, the vascularity of the injection site, and patient-specific risk factors may also contribute to the likelihood of toxicity [5,6].

Early clinical manifestations of LAST may include perioral and lingual numbness, a metallic taste, dizziness, and tinnitus. As toxicity progresses, agitation, altered consciousness, respiratory depression, loss of consciousness, and seizures may develop [7]. Cardiovascular manifestations may progress to cardiac conduction disturbances, arrhythmias, hypotension, and cardiac arrest [8]. Because clinical signs do not necessarily occur in the same sequence in every case, early recognition of LAST and prompt initiation of appropriate management are essential.

Intravenous lipid emulsion is one of the rescue therapies recommended particularly for severe cases of LAST [9]. The chylomicron-like structure of lipid emulsions is thought to reduce the circulating free fraction of lipophilic local anesthetics, thereby facilitating redistribution of the toxic agent from cardiac and central nervous system tissues into the plasma. This pharmacokinetic mechanism has been reported to contribute to treatment, particularly in cases involving highly lipophilic agents [10]. The effects of lipid emulsion are not considered to be limited solely to sequestration of the toxic agent; mechanisms such as support of myocardial contractility and the improvement of energy metabolism may also contribute to cardiovascular recovery [11,12].

The American Society of Regional Anesthesia and Pain Medicine (ASRA) emphasizes the importance of cardiopulmonary resuscitation and lipid emulsion therapy in the management of cardiac arrest associated with LAST. Similarly, the 2020 American Heart Association (AHA) Guidelines for Cardiopulmonary Resuscitation and Emergency Cardiovascular Care state that, in addition to standard resuscitation measures, administration of intravenous lipid emulsion may be reasonable in cases of LAST, particularly in cardiac arrest caused by bupivacaine toxicity [13,14].

In contrast, previous survey-based studies have reported limited knowledge of LAST and lipid emulsion therapy among dentists [15,16]. However, data simultaneously evaluating dentists’ self-reported knowledge and clinical awareness of LAST, their preventive practices, their knowledge of lipid emulsion therapy, and institutional availability remain limited. Therefore, this study aimed to comprehensively assess the knowledge and clinical awareness of LAST, preventive practices, knowledge of lipid emulsion therapy, and institutional availability among dentists actively practicing in Türkiye, and to examine the demographic and professional factors associated with knowledge level.

## 2. Materials and Methods

### 2.1. Study Design and Questionnaire Development

This descriptive cross-sectional study was conducted online. Data were collected using a structured 15-item questionnaire developed by the researchers through the Google Forms platform (Google LLC, Mountain View, CA, USA). The questionnaire items were developed with reference to previous studies evaluating knowledge and awareness of LAST and lipid emulsion therapy [15,17], the 2020 ASRA LAST checklist [9] and the recommendations included in the 2020 AHA Guidelines for Cardiopulmonary Resuscitation and Emergency Cardiovascular Care [14].

The questionnaire consisted of two sections and was administered to participants in Turkish. The first section collected information on participants’ demographic and professional characteristics, including age, sex, year of graduation, professional status, specialty, and type of employing institution. The second section included closed-ended questions assessing self-reported knowledge of LAST, whether the label or contents of the local anesthetic were checked, knowledge of the maximum recommended dose, previous experience with a case of LAST, preventive practices related to LAST, knowledge of lipid emulsion therapy, and the availability of lipid emulsion at the participants’ institutions.

Because the questionnaire comprised independent items assessing distinct domains of knowledge, clinical practice, and institutional availability, and was not designed as a unidimensional scale, neither a total score nor an internal consistency coefficient was calculated. No formal psychometric assessment of validity and reliability, expert panel review, or pilot testing was conducted before data collection. This issue was acknowledged as a limitation of the study.

### 2.2. Sample Size Calculation

The sample size was calculated before data collection based on a single-population proportion. In a study conducted among dentists by Oksuz et al., 67.3% of participants were reported to have no knowledge of lipid emulsion therapy [15]. Based on this proportion, the minimum required sample size was determined to be 339 participants, assuming a 95% confidence level and a 5% margin of error. The final analyses were conducted using data from 369 participants.

### 2.3. Participants and Data Collection

The survey link was distributed by the researchers via WhatsApp (WhatsApp LLC, Menlo Park, CA, USA) to dentists actively practicing throughout Türkiye. Data were collected online between 28 June and 23 September 2025.

Participants’ eligibility was assessed based on the education and employment information they reported in the questionnaire. Dental students and individuals who reported that they were not actively practicing dentistry in Türkiye were excluded. Questionnaires with at least one unanswered item required for the analysis were considered incomplete. Potential duplicate records were identified by reviewing forms that showed exact matches across demographic and professional variables and all questionnaire responses, together with their submission timestamps; only the first record from each duplicate submission was retained for analysis.

**Figure 1:**
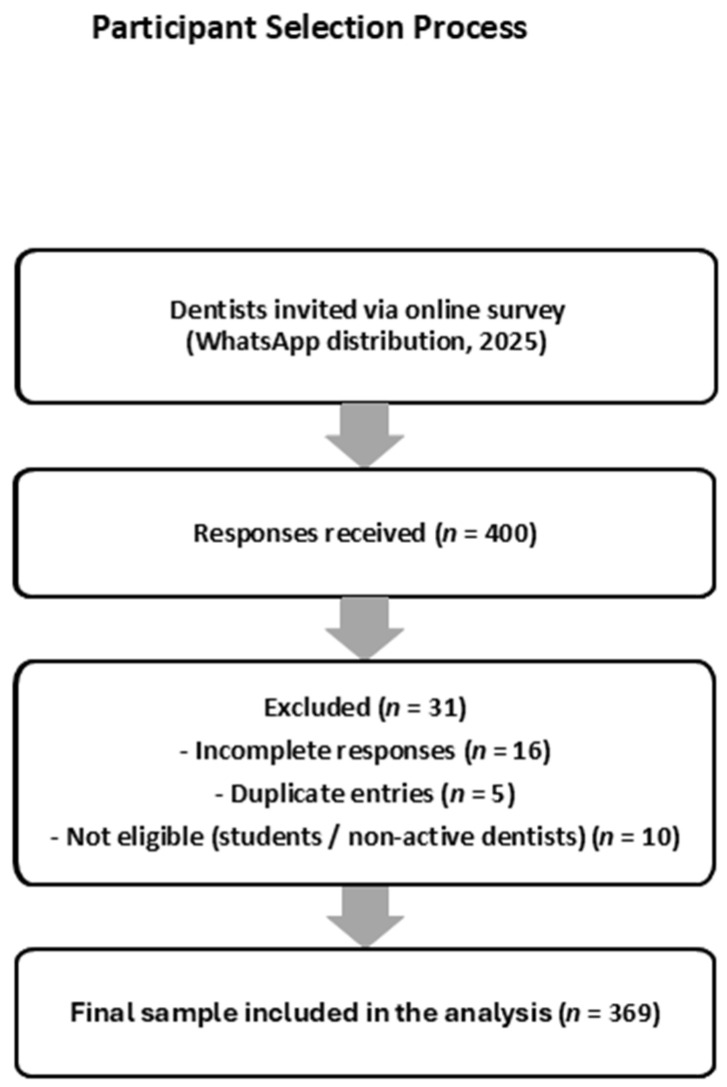
Flowchart of participant selection and inclusion in the study.

A total of 400 questionnaire forms were received. During data screening, 16 incomplete forms, 5 potential duplicate records, and 10 forms from participants who did not meet the inclusion criteria were excluded. Accordingly, the final analyses were conducted using data from 369 participants (Figure 1).

#### 2.3.1. Ethical Approval

The study protocol was approved by the Clinical Research Ethics Committee of the Faculty of Dentistry, Dicle University (Decision No. 2025-48; 25 June 2025). Participation was voluntary, and participants were informed about the purpose and scope of the study. They were not permitted to proceed to the subsequent survey items unless they had approved the Informed Consent Form presented at the beginning of the questionnaire. No personally identifiable information, such as names, surnames, or IP addresses, was requested, and all data were collected anonymously. Participants had the right to withdraw from the study before completing the questionnaire.

#### 2.3.2. Research Hypotheses

The following hypotheses were tested in this study:

1. Dentists’ knowledge of lipid emulsion therapy differs according to professional status.
2. Years of professional experience are associated with knowledge of lipid emulsion therapy.
3. Knowledge of lipid emulsion therapy differs according to the type of employing institution

#### 2.3.3. Statistical Analysis

Statistical analyses were performed using IBM SPSS Statistics for Windows, Version 31.0 (IBM Corp., Armonk, NY, USA). The analyses were conducted using complete data from 369 participants. Categorical variables were presented as frequencies and percentages. To provide a detailed description of the sample, professional status was reported using the original categories indicated by the participants in the descriptive analyses. For comparative and multivariable analyses, however, participants were classified into three groups: general dentists, research assistants, and specialists/academics. Dentists from all specialty fields, together with associate professors and professors, were combined into the specialists/academics group.

The Kruskal–Wallis test was used to compare self-reported knowledge levels regarding lipid emulsion therapy across professional status groups. When a statistically significant difference was identified, pairwise comparisons were performed using Dunn’s test, with Bonferroni adjustment applied to the *p*-values. Effect size was reported using the epsilon-squared (ε^2^) coefficient. The association between years of professional experience and knowledge level was evaluated using Spearman’s rank correlation analysis.

For the multivariable assessment of factors associated with knowledge level, multinomial logistic regression analysis was used because the proportional odds (parallel lines) assumption was not met. Knowledge of lipid emulsion therapy was presented using the five original response categories in the descriptive analyses. For the multinomial logistic regression analysis, these categories were combined into three groups—low knowledge, superficial knowledge, and basic/detailed knowledge—to reduce sparse cells and improve the stability of parameter estimates. The low-knowledge group comprised participants who had no knowledge or had only heard of the therapy, whereas the basic/detailed-knowledge group comprised those who reported basic knowledge or detailed knowledge of the administration protocol. The low-knowledge group was used as the reference outcome category.

In the multinomial logistic regression analysis, age was categorized as ≤34 years, 35–44 years, and ≥45 years; professional experience as 0–10 years, 11–20 years, and >20 years; and type of employing institution as public oral and dental healthcare institution, university, and private/other. Private clinics or dental offices, private hospitals, and other institutions were combined into the private/other category.

To reduce potential multicollinearity between age and professional experience, these variables were entered into separate models. Model 1 included professional experience, type of employing institution, and professional status, whereas Model 2 included age group, type of employing institution, and professional status. Regression results were reported as adjusted odds ratios (aORs), 95% confidence intervals, and *p*-values. The overall significance of the models was assessed using the likelihood-ratio chi-square test, their explanatory power using Nagelkerke’s pseudo-R^2^, and model fit using the Pearson and deviance goodness-of-fit tests. All tests were two-sided, and statistical significance was set at *p* < 0.05.

## 3. Results

### 3.1. Demographic Characteristics of the Participants

A total of 369 dentists were included in the study (Table 1).

The study group comprised 236 men (64.0%) and 133 women (36.0%). The largest age group consisted of dentists aged 25–34 years (*n* = 218, 59.1%), followed by those aged 35–44 years (*n* = 81, 22.0%). In addition, 24 participants (6.5%) were younger than 25 years. With respect to professional experience, the largest group consisted of dentists with 0–5 years of experience (*n* = 202, 54.7%).

When the detailed distribution of professional status was examined, general dentists constituted the majority of participants (*n* = 238, 64.5%). The sample also included 41 research assistants (11.1%), 88 dentists from various specialty fields (23.8%), and two academics holding the title of associate professor or professor (0.5%). For statistical comparisons, professional status was classified into three groups: general dentists (*n* = 238, 64.5%), research assistants (*n* = 41, 11.1%), and specialists/academics (*n* = 90, 24.4%).

Of the participants, 164 (44.4%) worked in a public hospital or oral and dental health center, 115 (31.2%) in a university hospital, and 74 (20.1%) in a private clinic or dental office. Six participants (1.6%) worked in a private hospital, while 10 (2.7%) worked in other institutions. No missing data were identified for the demographic and professional variables evaluated.

### 3.2. Local Anesthetic Practices and Awareness of LAST

**Figure 2:**
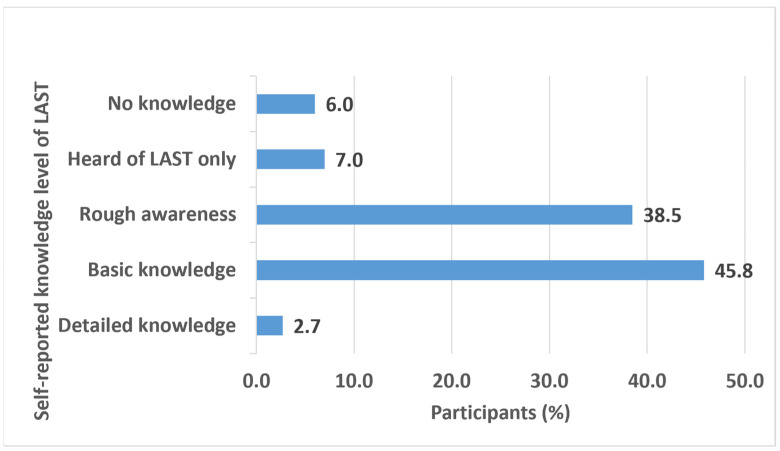
Dentists’ self-reported knowledge levels regarding local anesthetic systemic toxicity.

The distribution of participants’ self-reported knowledge levels regarding LAST is presented in Figure 2.

Detailed response distributions regarding local anesthetic practices and awareness of LAST are presented in Table 2.

The distribution of measures reported by participants to prevent LAST is presented in Table 3.

### 3.3. Knowledge of Lipid Emulsion Therapy and Institutional Availability

Among the participants, 32.2% reported having no knowledge of lipid emulsion therapy, while 26.8% stated that they had only heard of the therapy. Only 0.5% reported having detailed knowledge of the administration steps and dosing protocol. Regarding the availability of lipid emulsion at their institution, 45.0% reported that they did not know whether it was available, 40.9% stated that it was unavailable, and 14.1% indicated that it was available. Detailed distributions are presented in Table 4.

#### Comparison of Knowledge of Lipid Emulsion Therapy According to Professional Status

Participants’ knowledge levels regarding lipid emulsion therapy differed significantly across professional status groups (H(2) = 13.129; *p* = 0.001; ε^2^ = 0.030). In the Dunn–Bonferroni pairwise comparisons, only the difference between specialists/academics and general dentists was statistically significant (adjusted *p* = 0.003). No statistically significant differences were observed in the other pairwise comparisons (Table 5).

### 3.4. Association Between Professional Experience and Knowledge of Lipid Emulsion Therapy

The association between professional experience and knowledge of lipid emulsion therapy was evaluated using Spearman’s rank correlation analysis. No statistically significant association was found between the two variables (ρ = 0.020; *p* = 0.702; *n* = 369).

### 3.5. Factors Associated with Knowledge of Lipid Emulsion Therapy: Multinomial Logistic Regression Analysis

Factors associated with knowledge of lipid emulsion therapy were evaluated using two separate multinomial logistic regression models. In the analysis, 218 participants (59.1%) were classified in the low-knowledge group, 90 (24.4%) in the superficial-knowledge group, and 61 (16.5%) in the basic/detailed-knowledge group. In both models, the low-knowledge category was used as the reference outcome category.

Model 1, which included professional experience, type of employing institution, and professional status, was statistically significant compared with the intercept-only model (χ^2^(12) = 57.371; *p* < 0.001; Nagelkerke pseudo-R^2^ = 0.169). Likelihood-ratio tests indicated that professional experience (*p* = 0.036), type of employing institution (*p* = 0.016), and professional status (*p* < 0.001) were significantly associated with knowledge of lipid emulsion therapy.

Model 2, which included age group, type of employing institution, and professional status, was also statistically significant compared with the intercept-only model (χ^2^(12) = 64.559; *p* < 0.001; Nagelkerke pseudo-R^2^ = 0.189). In this model, age group (*p* = 0.002), type of employing institution (*p* = 0.031), and professional status (*p* < 0.001) were significantly associated with knowledge level. Category-specific adjusted odds ratios, 95% confidence intervals, and *p*-values are presented in Table 6.

For Model 1, the Pearson goodness-of-fit test was χ^2^(20) = 87.138 and the deviance goodness-of-fit test was χ^2^(20) = 100.109. For Model 2, the corresponding values were χ^2^(20) = 91.417 and χ^2^(20) = 101.584, respectively (all *p* < 0.001). In addition, zero-frequency cells were observed in some subgroups.

## 4. Discussion

LAST is a rare but potentially life-threatening complication. Prompt diagnosis and early intervention are critical for prognosis [18]. Because it can present with a wide range of clinical manifestations and many cases may go unreported, accurately determining the true incidence of LAST remains difficult [19]. In the present study, dentists’ knowledge and clinical awareness of LAST, their knowledge of lipid emulsion therapy, and the institutional availability of lipid emulsion were evaluated. The findings showed that participants’ awareness of LAST varied, whereas their knowledge of lipid emulsion therapy was largely concentrated in the low and superficial categories. In addition, 45.0% of participants reported that they did not know whether lipid emulsion was available at their institution, 40.9% stated that it was unavailable, and only 14.1% indicated that it was available.

These findings are generally consistent with those of previous studies conducted among different groups of healthcare professionals and dentists [15,16,20]. Given the limited number of studies addressing this issue specifically within dentistry in Türkiye, the present findings suggest that education on LAST and institutional emergency preparedness may need to be reviewed.

During the inferior alveolar nerve block, a procedure commonly performed in dentistry, the rate of positive aspiration has been reported to be as high as 15.3% [21]. This finding underscores the importance of aspiration before administration of the anesthetic solution and of implementing measures to prevent intravascular injection. The literature also recommends calculating the maximum local anesthetic dose in advance and using a slow injection technique with aspiration to reduce the risk of LAST [22,23].

In the present study, the most frequently reported preventive practices were obtaining a history of systemic diseases and medication use (74.8%), using a slow injection technique with aspiration (69.1%), and preferring infiltration anesthesia over regional anesthesia when appropriate (53.7%). In contrast, only 40.1% of participants reported calculating the local anesthetic dose according to the patient’s body weight. While 18.7% stated that they had no knowledge of LAST or did not take any preventive measures, only 3.3% reported having prepared an emergency response plan, and 2.2% indicated that they were prepared to use treatment options such as lipid emulsion. These findings suggest that some basic preventive practices are relatively common; however, further improvement is needed in dose calculation and emergency preparedness.

To facilitate prompt intervention in severe cases of LAST, it is recommended that 20% intravenous lipid emulsion be readily available in clinical settings where local anesthetics are administered [24,25]. The 2020 ASRA LAST checklist includes the administration of 20% lipid emulsion according to specified bolus and infusion protocols as part of LAST management [9]. In contrast, only 0.5% of participants in the present study reported having detailed knowledge of the administration steps and dosing protocol. This finding suggests that current recommendations for LAST management may have been only partially incorporated into dental practice.

In the study conducted by Oksuz et al., 51% of participants were reported to know the maximum doses of local anesthetics, 67.3% had no knowledge of lipid emulsion therapy, and only 1.5% knew how the treatment should be administered [15]. Similarly, Karasu et al. reported that 59.4% of dentists had never heard of lipid emulsion therapy and that only 2.1% had knowledge of the treatment [16]. Although the proportion of participants reporting no knowledge of lipid emulsion therapy appeared lower in the present study than in previous studies, detailed knowledge of the administration steps and dosing protocol remained limited. Because the response options, sample characteristics, and data collection periods differed across studies, this finding should not be interpreted as evidence that awareness has increased over time. The findings suggest that awareness of lipid emulsion therapy does not necessarily indicate adequate knowledge of its administration steps and dosing protocol.

Deficiencies in knowledge and awareness of LAST have been reported not only among dentists but also among other groups of healthcare professionals [17,26]. In a study conducted by Surani et al. among 134 resident physicians in Malaysia, awareness of LAST increased from 16.4% before training to 97.8% after a brief educational intervention [17]. Although this finding suggests that targeted education may improve awareness of LAST, direct comparison with the present results is limited because of differences in study populations and assessment methods. In another study conducted among emergency physicians in South Africa, only 25% of participants reported access to lipid emulsion in their clinical setting, while 33% knew the correct administration protocol [26]. These findings indicate that shortcomings in knowledge and institutional preparedness regarding LAST management may also be present across different clinical disciplines.

Case reports in the literature suggest that timely administration of intravenous lipid emulsion may contribute to clinical recovery in severe cases of LAST [27,28]. Park et al. reported rapid improvement in the level of consciousness following administration of 20% intravenous lipid emulsion in a case of lidocaine toxicity that developed after dental local anesthetic infiltration [27]. Similarly, in a pediatric case reported by Jermolajeite et al., cardiac arrest caused by inadvertent intravenous administration of levobupivacaine resolved after early initiation of lipid emulsion therapy in conjunction with standard resuscitation [28]. However, because these findings are based on case reports, they should not be interpreted as definitive evidence of treatment efficacy, but rather as evidence indicating the potential clinical importance of timely intervention.

Retrospective studies have also reported the occurrence of LAST following bupivacaine infiltration and have emphasized that highly lipophilic local anesthetics should be used with particular caution because of their potential for systemic toxicity [29,30]. In the present study, only 33.3% of participants reported checking the label or contents of the local anesthetic solution before every administration, whereas 47.7% stated that they did so only occasionally and 19.0% reported that they never did so. This distribution suggests that routine verification of the composition and dose of the agent used was not consistently adopted by all participants.

In addition to this overall pattern, we evaluated whether knowledge of lipid emulsion therapy varied according to professional status and experience. A statistically significant difference was observed among professional status groups; however, pairwise comparisons showed that only the difference between general dentists and specialists/academics was significant. Nevertheless, the small effect size (ε^2^ = 0.030) indicates that the practical magnitude of this difference was limited. The absence of significant differences between research assistants and the other groups further suggests that knowledge level cannot be explained by professional status alone. Spearman’s rank correlation analysis showed no statistically significant association between years of professional experience and knowledge of lipid emulsion therapy. In contrast, the overall contribution of professional experience was significant in the multivariable model in which experience was evaluated categorically. This discrepancy suggests that the association did not follow a simple monotonic pattern with increasing seniority and may vary according to the outcome category and adjustment for other variables included in the model.

In the multivariable analyses, age, professional experience, type of employing institution, and professional status were each generally associated with knowledge level in separate models. Using private/other institutions as the reference category, participants working in public oral and dental healthcare institutions and universities had lower odds of being in the superficial-knowledge group rather than the low-knowledge group. However, no statistically significant and consistent association was observed between institution type and the basic/detailed-knowledge category. Similarly, the category-specific associations for age, experience, and professional status did not show a consistent pattern across all knowledge levels. Therefore, these findings should not be interpreted as indicating that a particular age, experience, professional status, or institution group directly results in a higher or lower level of knowledge. In addition, the significant Pearson and deviance goodness-of-fit tests in both models suggested possible model–data misfit, while sparse and zero-frequency cells may have limited the stability of the estimates. For these reasons, the multivariable analysis results should be interpreted with caution.

The present findings suggest that strengthening practice-oriented education on LAST management and lipid emulsion therapy within dental curricula and continuing professional education programs may be beneficial. In addition, reviewing the availability of lipid emulsion, emergency response protocols, and staff preparedness in clinical institutions where local anesthetics are administered may contribute to patient safety. Future interventional studies should therefore examine the effects of education on LAST and lipid emulsion therapy on dentists’ knowledge, practical skills, and emergency preparedness using pretest–posttest assessments and long-term follow-up.

### Limitations

This study has several limitations. First, because the data were collected through a self-reported online questionnaire, participants’ responses may not fully reflect their actual knowledge or clinical practices. Similarly, reports regarding the availability of lipid emulsion at participants’ institutions were not independently verified against institutional records.

Second, no psychometric assessment of validity and reliability, expert panel review, or pilot testing was conducted for the researcher-developed questionnaire before data collection. Because the questionnaire consisted of independent items assessing different domains of knowledge, clinical practice, and institutional availability rather than functioning as a unidimensional scale, neither a total score nor an internal consistency coefficient was calculated.

Third, distributing the survey link through WhatsApp and relying on voluntary participation may have resulted in greater representation of dentists who were more active in professional communication groups. This sampling approach may have increased the risk of selection and response bias, thereby limiting the extent to which the sample represents all dentists in Türkiye. The administration of the questionnaire only in Turkish and the conduct of the study exclusively in Türkiye also restrict the generalizability of the findings to different linguistic, cultural, and healthcare system contexts.

The cross-sectional design of the study does not allow temporal or causal relationships to be established between the demographic and professional characteristics examined and knowledge level. In addition, combining dentists from different specialty fields and academics into a single specialist/academic group may have masked potential differences among these subgroups. Finally, the small number of participants or absence of observations in some category combinations in the multinomial logistic regression models, together with the statistically significant Pearson and deviance goodness-of-fit tests, may have limited model fit and the stability of the adjusted estimates.

## 5. Conclusions

This study showed that dentists’ self-reported awareness of LAST varied and that detailed knowledge of the administration steps and dosing protocol for lipid emulsion therapy remained limited. The substantial proportion of participants who either did not know whether lipid emulsion was available at their institution or reported that it was unavailable suggests that institutional emergency preparedness may need to be strengthened.

These findings indicate that enhancing practice-oriented education on the prevention and management of LAST in dental curricula and continuing professional education programs may be beneficial. Regular review of lipid emulsion availability, emergency response protocols, and staff preparedness in clinical institutions where local anesthetics are administered may contribute to patient safety.

## Figures and Tables

**Table 1:** Demographic and professional characteristics of the participants (*n* = 369).

Variable	Characteristic	*n*	%
Gender			
	Male	236	64.0
	Female	133	36.0
Age group			
	<25 years	24	6.5
	25–34 years	218	59.1
	35–44 years	81	22.0
	45–54 years	31	8.4
	≥55 years	15	4.1
Professional experience			
	0–5 years	202	54.7
	6–10 years	44	11.9
	11–20 years	81	22.0
	>20 years	42	11.4
Professional status			
	General dentist	238	64.5
	Research assistant	41	11.1
	Oral and maxillofacial surgery specialist	7	1.9
	Endodontics specialist	12	3.3
	Oral and maxillofacial radiology specialist	4	1.1
	Pediatric dentistry specialist	12	3.3
	Periodontology specialist	11	3.0
	Prosthodontics specialist	20	5.4
	Restorative dentistry specialist	22	6.0
	Academic (associate professor/professor)	2	0.5
Institution of Employment			
	Public hospital/oral and dental health center	164	44.4
	University hospital	115	31.2
	Private clinic/dental office	74	20.1
	Private hospital	6	1.6
	Other	10	2.7

Note: For comparative and multivariable analyses, professional status was categorized into three groups: general dentists (*n* = 238, 64.5%), research assistants (*n* = 41, 11.1%), and specialists/academics (*n* = 90, 24.4%). Percentages may not total 100.0 because of rounding.

**Table 2:** Distribution of participant responses regarding local anesthetic practices and LAST awareness (*n* = 369).

Survey Question	Response Option	*n*	%
Self-reported knowledge level regarding LAST	No knowledge	22	6.0
	Heard of LAST only	26	7.0
	Rough awareness without detailed knowledge	142	38.5
	Basic knowledge	169	45.8
	Detailed knowledge, including management steps	10	2.7
Do you check the label or contents of the local anesthetic before each administration?	Always	123	33.3
	Sometimes	176	47.7
	No	70	19.0
Do you know the maximum recommended dose of the local anesthetic you use?	Yes	144	39.0
	Partially	195	52.8
	No	30	8.1
Have you ever encountered a case of LAST?	Yes	63	17.1
	No	306	82.9

**Table 3:** Distribution of measures reported by participants to prevent LAST (multiple responses; *n* = 369).

Reported Measures to Prevent LAST	*n*	%
Obtaining a history of systemic diseases and medication use	276	74.8
Using a slow injection technique with aspiration	255	69.1
Preferring infiltration anesthesia over regional anesthesia when appropriate	198	53.7
Calculating the local anesthetic dose according to the patient’s body weight	148	40.1
Having no knowledge of LAST/taking no preventive measures	69	18.7
Other	15	4.1
Preparing an emergency response plan for the possible occurrence of LAST	12	3.3
Being prepared to use treatment options such as lipid emulsion	8	2.2

Because participants could select more than one response, the percentages exceed 100%. Percentages were calculated based on the total number of participants (n = 369).

**Table 4:** Participants’ self-reported knowledge of lipid emulsion therapy and its availability in their institutions (*n* = 369).

Survey Question	Response Option	*n*	%
How would you evaluate your level of knowledge regarding lipid emulsion therapy?	No knowledge	119	32.2
	Only heard of it	99	26.8
	Superficial knowledge	90	24.4
	Basic knowledge	59	16.0
	Detailed knowledge of the administration steps and dosing protocol	2	0.5
Is lipid emulsion available in the institution where you work?	I do not know	166	45.0
	No, it is not available	151	40.9
	Yes	52	14.1

Percentages may not total 100.0% due to rounding.

**Table 5:** Comparison of knowledge levels regarding lipid emulsion therapy according to professional status (*n* = 369).

Panel A. Mean Ranks of the Professional Status Groups		
Professional Status	*n*	Mean Rank
General dentist	238	170.63
Research assistant	41	209.12
Specialist/academic	90	212.02
Panel B. Dunn–Bonferroni post hoc pairwise comparisons		
Pairwise comparison	Standardized test statistic (z)	Adjusted *p*-value
General dentist–Research assistant	−2.215	0.080
General dentist–Specialist/academic	−3.254	0.003 *
Research assistant–Specialist/academic	−0.149	1.000

Kruskal–Wallis test: H(2) = 13.129; *p* = 0.001; ε^2^ = 0.030. ε^2^ denotes the effect size for the Kruskal–Wallis test. The p-values for pairwise comparisons were adjusted using the Bonferroni method. * Statistically significant (adjusted *p* < 0.05).

**Table 6:** Multinomial logistic regression analysis of factors associated with knowledge of lipid emulsion therapy (*n* = 369).

Panel A. Model Including Professional Experience			
Variable	Overall LR *p*-Value	Superficial Knowledge vs. Low Knowledge, aOR (95% CI); *p*	Basic/Detailed Knowledge vs. Low Knowledge, aOR (95% CI); *p*
Professional experience	0.036		
0–10 years		0.345 (0.149–0.801); *p* = 0.013	0.320 (0.114–0.898); *p* = 0.030
11–20 years		0.286 (0.114–0.720); *p* = 0.008	0.448 (0.154–1.297); *p* = 0.139
>20 years		Reference	Reference
Type of employing institution	0.016		
Public oral and dental healthcare institution		0.350 (0.174–0.702); *p* = 0.003	0.549 (0.214–1.409); *p* = 0.213
University		0.281 (0.104–0.760); *p* = 0.012	0.869 (0.251–3.014); *p* = 0.825
Private/other		Reference	Reference
Professional status	<0.001		
General dentist		0.240 (0.105–0.547); *p* < 0.001	0.395 (0.142–1.098); *p* = 0.075
Research assistant		0.134 (0.028–0.633); *p* = 0.011	1.812 (0.737–4.455); *p* = 0.196
Specialist/academic		Reference	Reference
Panel B. Model including age group			
Variable	Overall LR *p*-value	Superficial knowledge vs. low knowledge, aOR (95% CI); *p*	Basic/detailed knowledge vs. low knowledge, aOR (95% CI); *p*
Age group	0.002		
≤34 years		0.272 (0.121–0.612); *p* = 0.002	0.397 (0.144–1.095); *p* = 0.074
35–44 years		0.158 (0.062–0.400); *p* < 0.001	0.331 (0.111–0.983); *p* = 0.047
≥45 years		Reference	Reference
Type of employing institution	0.031		
Public oral and dental healthcare institution		0.358 (0.178–0.719); *p* = 0.004	0.675 (0.269–1.694); *p* = 0.402
University		0.290 (0.105–0.798); *p* = 0.017	0.872 (0.252–3.012); *p* = 0.828
Private/other		Reference	Reference
Professional status	<0.001		
General dentist		0.244 (0.105–0.568); *p* = 0.001	0.371 (0.134–1.029); *p* = 0.057
Research assistant		0.136 (0.029–0.648); *p* = 0.012	1.711 (0.692–4.232); *p* = 0.245
Specialist/academic		Reference	Reference

Model summary: Likelihood-ratio χ^2^(12) = 57.371; *p* < 0.001; Nagelkerke pseudo-R^2^ = 0.169. Model summary: Likelihood-ratio χ^2^(12) = 64.559; *p* < 0.001; Nagelkerke pseudo-R^2^ = 0.189. Notes: The dependent variable was evaluated in three categories. The “low knowledge” category was created by combining the responses “no knowledge” and “heard of it only,” whereas the “basic/detailed knowledge” category was created by combining the responses “basic knowledge” and “detailed knowledge of the protocol.” In both models, the low-knowledge group was used as the reference outcome category. The reference categories for the independent variables were >20 years of professional experience, age ≥45 years, private/other institution, and specialist/academic status. The aORs were mutually adjusted for the other variables included in the corresponding model. To reduce conceptual overlap and potential multicollinearity between age and professional experience, these variables were not entered into the same model and were instead evaluated in separate models. The overall LR p-value refers to the likelihood-ratio test assessing the joint contribution of all categories of the corresponding variable to the model. Because sparse cells were present and the Pearson and deviance goodness-of-fit tests were statistically significant, the findings should be interpreted with caution. aOR: adjusted odds ratio; CI: confidence interval; LR: likelihood ratio.

## Data Availability

The datasets generated and analyzed during the current study are not publicly available because they contain individual-level survey responses and demographic/professional information that may allow indirect identification of participants, particularly in small subgroups. However, anonymized data are available from the corresponding author upon reasonable request and in accordance with institutional ethical requirements.
